# Mitigation of COVID-19 clusters in Malaysia

**DOI:** 10.7189/jogh.10.0203105

**Published:** 2020-12

**Authors:** Monica Danial, Ann Lisa Arulappen, Alan Swee Hock Ch’ng, Irene Looi

**Affiliations:** 1 Clinical Research Center, Seberang Jaya Hospital, Ministry of Health Malaysia, Penang, Malaysia; 2 Pharmacy Department, Seberang Jaya Hospital, Penang, Malaysia; 3 Medical Department, Seberang Jaya Hospital, Penang, Malaysia

The abrupt emergence of the novel coronavirus has led to the efforts of all nations in the world to contain and slow down the progression and infection rate in their respective countries. A coronavirus cluster emerges when a number of infections occur simultaneously in the same location [1]. As of 16 September 2020, there were about 91 (10 active and 81 inactive) COVID-19 clusters in Malaysia. A COVID-19 cluster will be considered inactive if no new case were reported within 28 days. The top 5 clusters with more than 200 positive cases reported were Tabligh cluster, Depo Tahanan Imigresen Bukit Jalil (DTI Bukit Jalil) cluster, Benteng Lahad Datu (Benteng LD) cluster, Pedas cluster and Pesanteran cluster. Only the Benteng Lahad Datu cluster remains active among these clusters, with the number of cases reportedly increasing [2]. **Figure 1******illustrates the number of positive cases based on nationality for the top 5 clusters reported in Malaysia up to 16 September 2020. A series line shows the number of deaths associated with the respective clusters.

**Figure 1:**
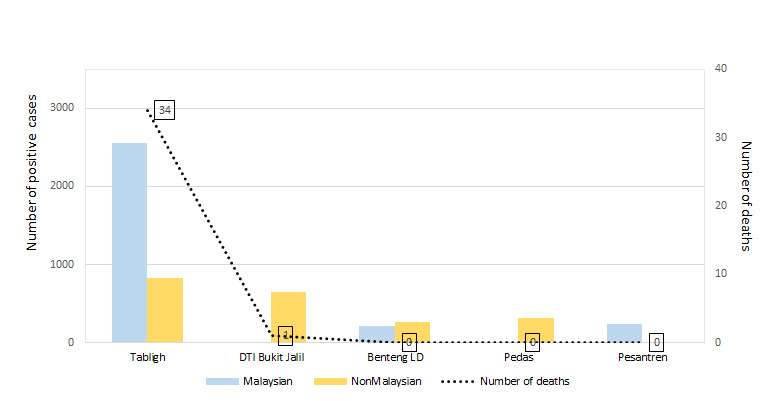
Primary COVID-19 clusters in Malaysia with reported more than 200 positive cases. The Tabligh and Pesantren clusters consist of higher number of Malaysians tested positive for COVID-19. On the contrary, in the DTI Bukit Jalil, Benteng LD and Pedas clusters, higher numbers of non-Malaysians tested positive for COVID-19. The Tabligh cluster had recorded the highest number of deaths of all the clusters. Data reported and tabulated as of 16 September 2020 [2,3].

## THE CLUSTERS WITH MORE THAN 200 POSITIVE CASES

The Tabligh cluster was linked to a religious gathering, which caused a massive increase in positive cases in Malaysia and abroad. The index case from this cluster was reported on 11 March 2020 in Malaysia. The number of cases grew rapidly and, with a view to control the spread within the population, the Movement Control Order (MCO) was enforced on 16 March 2020 [3]. Enforcing social distancing was the main goal of the MCO. These include bans on all mass gatherings, including religious, recreational, social and cultural activities. The closing of all educational institutions, schools and kindergartens was also ordered. Malaysian borders have been closed in addition to restrictions on travel between states, in particular to areas that have been designated coronavirus-affected areas or red zones. Only critical services were allowed to operate, and only one person per family was allowed to buy daily necessities [4]. The Tabligh cluster was confirmed to be inactive on 8 July 2020 with a total of 3375 positive cases identified [3].

The DTI Bukit Jalil cluster was mainly constituted of illegal immigrants who had been in contact with a COVID-19 positive case. The cluster was reported on 21 May 2020 and ended on 8 September 2020. As an initiative to contain the spread, the immigrants were retained at an immigration depo which were equipped with medical professionals, health facilities, timely meals up to 4 times per day and other necessities [5]. This measure has successfully curbed virus transmission among illegal immigrants and also Malaysians. About 653 positive cases were reported from this cluster [3].

The Benteng LD cluster originated from Sabah (West Malaysia). It is currently an active cluster with the index case reported on 1 September 2020. To date, about 472 positive cases have been identified from this cluster and their numbers have been reported to increase. This cluster's origin was traced back to the arrests of two illegal immigrants who were held in police custody at the Lahad Datu Police Headquarters. As a way of preventing the spread of the virus, public involvement in recreational activities in public parks and mass meetings were banned. In addition, the police have set up roadblocks in many areas of 'Ops Benteng' as an attempt to limit movement between locals and also to deter illegal migrants from entering Malaysia [6].

The Pedas cluster involving a chicken processing plant in Negeri Sembilan lasted from 28 June 2020 to 27 July 2020 with a total of 326 positive cases recorded [3]. Similar to the DTI Bukit Jalil cluster, the majority of the cases from the Pedas cluster were non-Malaysians. The transmission of Covid-19 among foreigners was directly linked to the overcrowded and congested living conditions of foreign workers’ residences. Therefore, in order to prevent further spread of the infection, the workers involved in this cluster were isolated and placed in quarantine centers for at least 14 days until no new cases were detected [7].The quarantine centers were equipped with similar facilities as the immigration depot.

Pesantren cluster was caused by Malaysian students returning back from a religious school in East Java, Indonesia. Positive cases from the cluster were initially reported on 16 April 2020. A total of 238 positive cases were identified from this cluster [3]. The initial screening and detection at the country entry points made it possible to successfully control the spread. All the students were quarantined after arriving from Indonesia at Kuala Lumpur International Airport (KLIA) [8]. On 20 June 2020, the cluster ended, with no reported new case.

**Figure:**
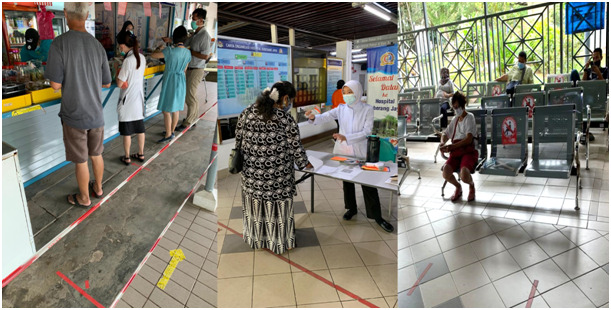
Photo: Malaysians adhering to the standard operating procedure (SOP) guidelines outlined by the Ministry of Health (MOH) Malaysia in maintaining physical distances and adorning face masks in public areas (photo collection by the authors).

## SUPER SPREADER INDEX CASE

The PUI Sivagangga cluster is another prominent cluster in Malaysia. Although there were no more than 200 positive cases reported from this cluster, this cluster gave rise to several sub-clusters in the Northern Region of Malaysia [3]. The index case for this cluster was a Malaysian permanent resident (PR) that had returned from Sivagangga, India. The spread in the local community was attributed to the failure of the index case to comply with the quarantine order after his return to Malaysia. Moreover, the index case was a confirmed carrier of the super spreader virus strain D614G. Localized Enhanced MCO (EMCO) was implemented thereafter with immediate closure of a few schools and offices as a way to localize and contain the spread. Also, strict adherence to the SOPs (Standard Operating Procedures) outlined by the Ministry of Health of Malaysia (MOH, Malaysia), was observed [9] in the affected areas.

## CONCLUSION

Early detection, containment and movement control played a crucial role in the mitigation efforts and reduced infection in the population.

Major COVID-19 transmission in Malaysia was prevented due to vigorous screening and identification at various entry points and illegal routes. Furthermore, localized EMCO was implemented to control and manage local transmission particularly in red zones. Moreover, law enforcement officers will routinely track the publics' strict adherence to the SOP guidelines illustrated by the MOH, Malaysia. Public adherence in maintaining physical distances from one another and practice of correctly wearing facemask will be checked by the authorities by doing regular inspections in public areas, business premises, and in the public and private vehicles. Those who did not adhere to the SOPs will be fined as minimum as MYR1000 (US$244) and in some cases faces imprisonment. Health authorities, law enforcement officers, volunteers and the public work closely and hand in hand around the clock to prevent COVID-19 transmission. ‘Work from home’, wearing mask and frequent hand washing cum sanitizing, physical and social distancing became the new normal.

## References

[1] What does coronavirus cluster mean? COVID-19 terms explained. 2020. Available: https://www.aljazeera.com/news/2020/03/coronavirus-terminology-explained-covid-19-glossary-200323064432820.html. Accessed: 18 September 2020.

[2] Covid-19 in Malaysia. 2020. Available: https://newslab.malaysiakini.com/covid-19/en/clusters. Accessed: 18 September 2020.

[3] Covid-19 Updates [Internet]. Crisis Preparedness & Response Center (CRRC) Ministry of Health Malaysia. 2020. Available from: https://www.facebook.com/CPRCKebangsaanKKM/posts/1442751895917490. Accessed: 18 September 2020.

[4] Covid-19: Movement Control Order imposed with only essential sectors operating. 2020. Available: https://www.nst.com.my/news/nation/2020/03/575177/covid-19-movement-control-order-imposed-only-essential-sectors-operating. Accessed: 18 September 2020.

[5] PATI di Depot Tahanan Imigresen dijaga, diurus dengan baik. 2020. Available: https://www.bharian.com.my/berita/nasional/2020/05/687977/tangkap-pendatang-asing-tanpa-izin-dilakukan-sepanjang-tahun. Accessed: 9 September 2020.

[6] Sabah enforcing 'control measures' to manage Covid-19 outbreak. 2020. Available: https://www.nst.com.my/news/nation/2020/09/625505/sabah-enforcing-control-measures-manage-covid-19-outbreak. Accessed: 23 September 2020.

[7] Pedas Cluster Started By Malaysians Who Infected Foreign WorkersMOH. 2020 Available: https://codeblue.galencentre.org/2020/06/22/pedas-cluster-started-by-malaysians-who-infected-foreign-workers-moh/. Accessed: 17 September 2020.

[8] Health Ministry declares end of Malaysia's fourth largest Covid-19 cluster dubbed ‘Pesantren’. 2020. Available: https://www.msn.com/en-my/news/national/health-ministry-declares-end-of-malaysias-fourth-largest-covid-19-cluster-dubbed-pesantren/ar-BB15KLNM?li=BBr8Hnu. Accessed: 23 September 2020.

[9] Kluster PUI. Sivagangga. 2020. Available: https://www.facebook.com/kementeriankesihatanmalaysia/posts/keputusan-sama-ada-kluster-pui-sivagangga-adalah-super-spreader-hanya-diketahui-/10157289227816237/. Accessed: 15 September 2020.

